# OA04.01. Effects of relaxation response intervention on endogenous progenitor cells in a hypertensive population

**DOI:** 10.1186/1472-6882-12-S1-O13

**Published:** 2012-06-12

**Authors:** A Fyfe-Johnson, D Taylor, C Zierold, J Dusek

**Affiliations:** 1 Penny George Inst. for Health & Healing, ANW, Allina Health, Minneapolis, USA; 2 Center for Cardiovascular Repair, University of Minnesota, Minneapolis, USA

## Purpose

To examine the impact of a relaxation response intervention on endogenous progenitor cell populations in a systolic hypertension population.

## Methods

These ancillary data are an extension of a current study investigating the impact of a relaxation response meditation (RR) in a systolic hypertension population. Data were collected from 15 subjects with systolic hypertension: before and after an 8-week manualized RR meditation intervention, and compared to subjects receiving health education. Primary outcomes were pre and post-progenitor cell populations measured by flow cytometry (FACScan). After lysis and processing, cell number and viability were determined using an automated Guava ViaCount assay counter, Millipore. FACScan raw data were collected using LSRII Cytometer, BD Biosciences. Paired T-tests were used to test for significant differences in mean pre and post progenitor cell changes. Cell surface markers of interest were CD45, CD34, CD31, AC133, CXCR4, and VEGFR2.

## Results

Progenitor cells offer the potential to be a meaningful outcome measure for integrative medicine, and are specifically relevant to cardiovascular disease. Arterial repair has powerful clinical applications; particularly for hypertension. Relative to health education control, we found a marked increase in endogenous progenitor cells expressing CD45, CD34, CD31, CXCR4, and VEGFR2 (p<0.01) after 8 weeks of RR meditation. The largest increase was seen in the CD34 subpopulation (40.1%, p<0.05). A significant decrease was shown in cells expressing AC133 (61.1%, p<0.05). In concordance with an increase in the CD34 subpopulation, these data suggest that increases in progenitor cells are evident in the middle of the differentiation process and offer a promising signal for the prospect of arterial repair with mind/body interventions.

## Conclusion

Based on preliminary data, RR meditation appears to increase populations of endogenous progenitor cells in patients with systolic hypertension. These findings provide incentive to further investigate endogenous progenitor cells as a novel outcome measure for mind/body therapies.

